# Medication non-adherence in acute coronary syndrome patients in Duhok, Iraqi Kurdistan

**DOI:** 10.1097/MS9.0000000000002790

**Published:** 2025-01-30

**Authors:** Ameen M. Mohammad, Asmaa M. Sulaiman, Kawa F. Dizaye

**Affiliations:** a Department of Internal Medicine, College of Medicine, University of Duhok, Kurdistan Region, Iraq; b Fellow of Clinical Pharmacy, Kurdistan Board of Medical Specializations, Erbil, Kurdistan Region, Iraq; c College of Medicine, Hawler Medical University, Kurdistan Region, Iraq

**Keywords:** acute coronary syndrome, adherence to medications, Eastern Mediterranean

## Abstract

**Background:**

Adherence to long-term secondary preventive therapies is vital for improving outcomes in patients recovering from acute coronary syndrome (ACS). This study aims to quantify the extent of non-adherence to these therapies in Iraq and identify the main factors contributing to this issue, addressing a research gap in the Eastern Mediterranean region.

**Methods:**

This cross-sectional study was conducted from June 2023 to March 2024 in Cardiology Ward of Azadi Teaching Hospital and the Duhok Heart Center, Duhok, Kurdistan Region of Iraq, enrolling 400 patients diagnosed with (ACS). The Adherence in Chronic Diseases Scale (ACDS) was utilized to categorize patients into high, intermediate, or low adherence groups. The study questionnaire comprised three sections including clinicodemographic data, adherence assessment based on the ACDS, and patient-reported reasons for non-adherence.

**Results:**

The study revealed that the mean age of the participants was 65.78 ± 11.8 years. Within the sample, 24% reported low adherence, 39% reported medium adherence, and only 37% exhibited high adherence. Significant associations were observed between low adherence and older age (*P* = 0.026), lower education levels (*P* = 0.0051), and the presence of endocrine disorders (*P* = 0.029). Conversely, higher adherence was found among patients taking 3–5 different medication classes (*P* = 0.0003) and those who underwent coronary interventions (*P* = 0.014). The primary reason for non-adherence was forgetfulness (89.5%).

**Conclusion:**

The study concludes that a substantial portion of ACS patients in Iraq show low adherence to secondary preventive therapies. To increase adherence among patients with ACS, strategies should be developed to improve medication adherence and promote healthy behaviors simultaneously, Forgetfulness and lack of follow-up are the primary reasons for non-adherence.

**HIGHLIGHTS**

- Only 37% of ACS patients exhibited high adherence to secondary pharmacological prevention.
- High adherence was observed in patients who underwent coronary interventions.
- Old age, lower educational level, and having endocrine disorders were associated with low adherence.
- Forgetfulness was the most common reason for non-adherence.

## Introduction

Patients who survive acute coronary syndrome (ACS) have a substantial risk of recurrent cardiovascular events[1]. Additionally, repeated episodes are associated with worse outcomes compared to initial events. Secondary preventive pharmacologic therapy is recommended following ACS to improve long-term patient outcomes^[2,3]^. According to the American College of Cardiology/American Heart Association guidelines, unless contraindicated, almost all patients recovering from ACS should initiate angiotensin-converting enzyme inhibitors or angiotensin II receptor blockers, beta-blockers, statins, and antiplatelet therapy for the long term. These therapies typically require lifelong or long-term treatment[4].

Medication nonadherence is a prevalent issue that leads to poor patient outcomes and significantly increases healthcare expenses[2]. According to WHO data, only about half of chronically ill individuals in wealthy nations strictly adhere to medication recommendations[5]. Nonadherence to optimal cardiovascular drugs is linked to a 30% increased risk of cardiovascular events in ACS patients[6]. Thus, improving adherence can reduce the clinical and economic burden on the healthcare system while enhancing patients’ quality of life[2].

Adherence is a multifaceted phenomenon influenced by various factors. Understanding the key characteristics that determine adherence among ACS patients can help physicians tailor their treatment approaches and implement effective hospital interventions to improve adherence and cardiovascular outcomes^[2,7]^. Patients’ medication-taking behaviors can be either intentional or unintentional. Unintentional nonadherence is often due to passive forgetfulness, while intentional nonadherence involves a deliberate decision to avoid taking medications. Consequently, the causes and potential solutions may differ depending on the type of nonadherence^[6,8]^.

Adherence can be assessed using both direct and indirect methods. Direct methods, such as therapeutic monitoring, are not widely used due to their high cost. Indirect methods, such as self-reported questionnaires, are simple, inexpensive, and straightforward. These methods allow healthcare professionals to gather information about the causes of nonadherence, enabling them to personalize interventions to change negative attitudes among patients and healthcare teams[9].

The Adherence in Chronic Diseases Scale (ACDS) is a recently developed and validated tool for assessing treatment adherence in patients with chronic illnesses. However, its application has been limited to a few patient groups[5]. Research on medication non-adherence among ACS patients in the Middle East is very scanty, and no studies have yet evaluated this issue in Iraq or the factors associated with non-adherence. This study therefore intends to utilize the ACDS questionnaire to evaluate the adherence and compliance to medications for secondary prevention in ACS and explore the primary determinants of factors influencing medication adherence.

## Materials and methods

### Study design and participants

This cross-sectional study was conducted, between 1 June 2023 and 1 March 2024, in the Cardiology Ward of Azadi Teaching Hospital and the Duhok Heart Center, located in Duhok province, Kurdistan Region of Iraq. The study enrolled a total of 400 patients diagnosed with ACS. Data were gathered through face-to-face interviews with the patients or, a family member when the patients were unable to complete the study questionnaire themselves. The study adhered strictly to guidelines reported by Strengthening the Reporting of cohort, cross-sectional and case-control studies in Surgery (STROCSS) criteria[10].

### Study measurement

ACDS was utilized for the study. This questionnaire comprised three sections; The first section included a clinicodemographic profile of the patients, which captured data on age, gender, residency, education level, economic status, employment, marital status, past medical histories, past medication histories, type of ACS, cardiovascular risk factors for ACS, and type of revascularization interventions at the time of enrollment.

The second section utilized a standardized ACDS questionnaire to measure adherence to treatment recommendations among chronically ill patients. The ACDS, was developed by A. Kubica and validated in individuals with coronary artery disease[11]. Two specialists in the field of cardiovascular diseases and with relevant experience assessed the questionnaire in terms of content validity and cross-cultural adaptation. A pilot testing was conducted on a population comprising 20 patients with ACS aimed to ascertain the internal consistency of the instrument and Cronbach alpha was 0.778. The questionnaire consists of seven questions assessing patients’ adherence to medications, each with five multiple-choice responses. Questions 1–5 address medication-related patient behavior, while questions 6 and 7 evaluate physician-patient interaction, which indirectly affects adherence. Each item on the scale receives 0–4 points based on the response. Adherence was assessed using the scoring system established in the validation article: a score of ≥27 indicates high adherence to treatment, scores of 21–26 indicate intermediate adherence, and scores ≤20 indicate low adherence.

The final section of the study questionnaire gathered patient opinions on the primary reasons for medication non-adherence, which were classified into categories such as lack of knowledge, complicated polypharmacy, non-affordability, non-availability, forgetfulness, becoming bored with medicines, improved health, fear of medication side effects, lack of follow-up, carelessness.

### Inclusion/exclusion criteria

Patients older than 18 years who were diagnosed with ACS were included in this study. Exclusion criteria were patients with no medication history, those who did not consent to participate, and questionnaires with incomplete information or missed data.

### Ethical approval

The final study protocol received approval from the Ethics and Scientific Committee of the Higher Council of Medical Specialization in the Kurdistan Region of Iraq on 12 April 2023 with a reference number of 844. Written informed consent was obtained from all patients upon enrollment. The study adhered strictly to the ethical standards outlined in the Declaration of Helsinki for medical research involving human subjects.

### Statistical analysis

All data were initially entered into Microsoft Excel for coding and cleaning purposes. Subsequently, the cleaned data were transferred to GraphPad Prism Version 10.2 for statistical analysis. Categorical variables were presented as frequencies and percentages. Continuous variables were demonstrated as mean and standard deviation. The Shapiro-Wilk test was conducted to assess the normality of the continuous variables, revealing a non-normal distribution. Consequently, nonparametric tests were employed to determine the significance between study variables. The Mann-Whitney test was used to compare two groups, while the Kruskal-Wallis test was applied for comparisons involving three or more groups. A *P* value of less than 0.05 was considered statistically significant.

## Results

### Sociodemographic characteristics

A total number of 400 ACS patients were included in the study. The mean age of our study population was 65.78 ± 11.8 years. Patients in the age group of 51–70 represented more than half of the study participants. About two-fifths of the patients were male. Most of the patients were illiterate (71.5 %), and only 1% had a university qualification. The majority of study patients had a middle income and were unemployed. About two-thirds were married. Regarding smoking status one-third of the study cohort were smokers and about half had a positive family history of cardiovascular dieases (CVDs). Table 1 shows the sociodemographic characteristics of the study patients.

**Table 1:** Sociodemographic characteristics and its association with ACDS results (n = 400)

Variables	n (%)	Mean ± SD	*P* value
Age			
31–50	41 (10.3)	22.66 ± 6.16	0.026
51–70	229 (57.3)	23.28 ± 4.88	
>70	130 (32.5)	22.18 ± 5.11	
Mean (SD)	65.78 (±11.8)	—	
Gender			
Male	165 (41.3)	22.55 ± 5.72	0.89
Female	235 (58.8)	23.08 ± 4.65	
Physical activity			
Sedentary	359 (89.8)	22.91 ± 5.07	0.99
Active	41 (10.3)	22.37 ± 5.59	
Education level			
Illiterate	286 (71.5)	22.90 ± 5.02	0.0051
Primary	78 (19.5)	21.82 ± 5.86	
Secondary	3 (0.8)	19.66 ± 4.03	
Vocational	29 (7.3)	25.38 ± 2.83	
Tertiary	4 (1.0)	24 ± 3	
Economic status			
Low income	48 (12.0)	23.23 ± 3.98	0.25
Middle income	348 (87.0)	22.77 ± 2.26	
High income	4 (1.0)	26.25 ± 1.3	
Employment status			
Employed	24 (6.0)	24.21 ± 3.84	0.12
Unemployed	351 (87.7)	22.71 ± 5.21	
Retired	25 (6.3)	23.68 ± 4.73	
Place of residence			
Urban	286 (71.5)	22.73 ± 5.25	0.52
Rural	114 (28.5)	23.19 ± 4.77	
Marital status			
Single	8 (2.0)	18.88 ± 8.88	0.15
Married	259 (64.8)	23.08 ± 5.09	
Widowed	133 (33.3)	22.65 ± 4.76	
Type of acute coronary syndrome			
STEMI	72 (18.0)	22.54 ± 6.11	0.45
NSTEMI	160 (40.0)	22.52 ± 5.21	
Unstable angina	168 (42.0)	23.32 ± 4.56	
Smoking			
Yes	131 (32.75)	22.77 ± 5.37	0.61
No	269 (67.25)	22.90 ± 5	
Family history of CVD			
Yes	215 (53.75)	23.05 ± 5.11	0.29
No	185 (46.25)	22.63 ± 5.13	

### Relationship of ACDS score and sociodemographic variables

**Figure 1.**
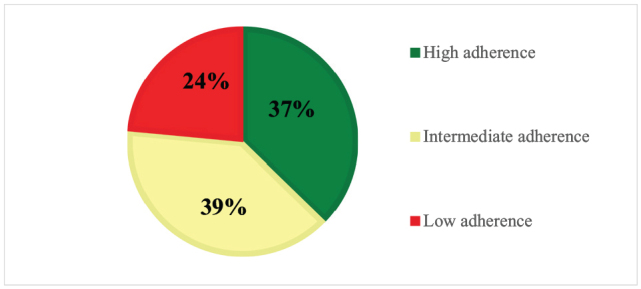
Adherence classification.

Figure 1 shows that 24% of our study sample had low adherence to the medications, and 39% and 37% of the patients had intermediate and high adherence to their medications, respectively. We found that patients in the age group of 51–70 and participants with vocational educational levels significantly had a higher mean of ACDS compared to their counterparts with *P* values of 0.026 and 0.0051, respectively. Females, those with high income, employed patients, married, and those with a positive family history had a better medication adherence mean. However, a significant association for these variables was not present. Table 1 presents the ACDS score and its association with sociodemographic variables.

### Relationship between ACDS score and clinical parameters

Our study revealed that patients with conditions such as hypertension, dyslipidemia, renal and liver diseases demonstrated relatively higher adherence to therapeutic recommendations. In contrast, patients with endocrine disorders like diabetes mellitus and obesity and cerebrovascular accidents exhibited lower adherence to medication recommendations. Notably, significant differences were observed only for endocrine disorders and lipid disorders.

Examining the ACDS scores in relation to medication types, a significant correlation was observed with adherence to anti-ischemic, lipid-lowering, and antihypertensive medication classes. No significant association was found for other medication classes. Patients taking three to five different medications had significantly higher ACDS scores compared to those taking higher than five medications, with a *P* value of 0.0003. Furthermore, patients who underwent percutaneous coronary intervention and coronary artery bypass grafting had significantly higher mean ACDS scores, measuring 23.41 ± 4.58 and 24.59 ± 2.85, respectively. Detailed results of the clinical parameters and their correlation with ACDS scores are presented in Table 2.

**Table 2:** Clinical parameters of patients and association with ACDS results (n = 400)

Variables	n (%)	Mean ± SD	*P* value
Comorbidities			
Hypertension			
Yes	330 (82.5)	23.13 ± 4.75	0.32
No	70 (17.5)	21.56 ± 6.47	
Endocrine disorders			
Yes	234 (58.5)	22.62 ± 4.94	0.029
No	166 (41.5)	23.19 ± 5.39	
Cerebrovascular accident			
Yes	41 (10.25)	22.39 ± 5.22	0.26
No	359 (89.75)	22.91 ± 5.13	
Dyslipidemia			
Yes	203 (50.75)	23.59 ± 4.35	0.014
No	197 (49.25)	22.11 ± 5.75	
Renal and liver disease			
Yes	79 (19.75)	22.87 ± 4.42	0.36
No	321 (80.25)	22.85 ± 5.30	
Malignancies			
Yes	15 (3.75)	24.6 ± 4.93	0.06
No	385 (96.25)	22.79 ± 5.13	
Medication classes			
Anti-Ischemic agents			
Yes	284 (71)	23.43 ± 4.71	0.0015
No	116 (29)	21.47 ± 5.83	
Lipid-lowering agents			
Yes	192 (48)	23.93 ± 4.06	0.0002
No	208 (52)	21.87 ± 5.79	
Anti-hypertensive agents			
Yes	341 (85.25)	23.32 ± 4.49	0.0074
No	59 (14.75)	20.17 ± 7.38	
Anti-diabetic agents			
Yes	194 (48.5)	23.07 ± 4.62	0.96
No	206 (51.5)	22.66 ± 5.57	
Anti-coagulant agents			
Yes	47 (11.75)	23.91 ± 4.53	0.15
No	353 (88.25)	22.72 ± 5.20	
Anti-arrhythmic agents			
Yes	26 (6.5)	23.58 ± 5.52	0.28
No	374 (93.5)	22.81 ± 5.11	
Chemotherapy			
Yes	14 (3.5)	24.64 ± 5.09	0.046
No	386 (96.5)	22.79 ± 5.13	
Number of medications			
≤2	137 (34.25)	21.63 ± 5.74	0.0003
3–5	(62.25)	23.74 ± 4.33	
>5	14 (3.5)	19.14 ± 7.77	
Interventions			
None	169 (42.25)	21.92 ± 5.85	0.014
Percutaneous coronary intervention	204 (51)	23.41 ± 4.58	
Coronary artery bypass grafting	27 (6.75)	24.59 ± 2.85	

### Reasons for non-adherent behaviors measured by ACDS

**Figure 2.**
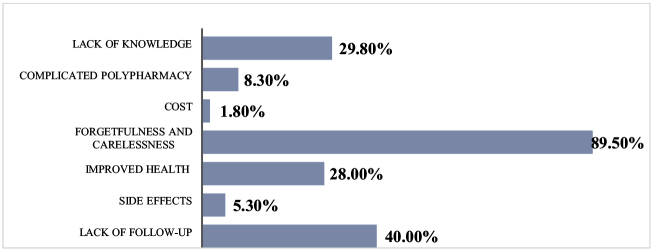
Reasons for non-adherence.

The ACDS results indicated that forgetfulness and carelessness were the most commonly reported reasons for patients’ non-adherence to their medications, accounting for 89.5% of cases. This was followed by a lack of follow-up (40%), lack of knowledge about their medications (29.8%), improved health (28%), complicated polypharmacy (8.3%), side effects (5.3%), and the least reported reason was cost (1.8%). Figure 2 illustrates the various reasons for non-adherence.

## Discussion

Patients with ACS who adhere strictly to their medications are at a lower risk of recurrence and complications. Therefore, this study aimed to evaluate the adherence of ACS patients to their secondary preventive medications. In this study, medication adherence was found to be poor among ACS patients, with 24% exhibiting low adherence and 39% showing intermediate adherence and only 37% have high adherence. According to our findings, patients in the middle aged groups, those with higher educational levels, patients with lipid disorders, those taking 3–5 medication classes, and those who underwent percutaneous coronary intervention (PCI) or coronary artery bypass grafting (CABG) were found to have significantly higher levels of adherence to the prescribed medications.

The mean age of patients in this study was 65.78 ± 11.8 years, which is higher than the mean age of patients with coronary artery disease reported from the same center[12]. A predominance of female patients was observed, with approximately 60% of the study population being female, a finding not consistent with other studies conducted at the same center^[13,14]^. Previous research from the Kurdistan Region of Iraq has identified traditional risk factors, such as smoking, dyslipidemia, diabetes mellitus, and hypertension, as predominant in ACS patients[15]. Our study observed a similar pattern.

The current study found that non-adherence to secondary pharmacological prevention was significantly higher in older patients. This finding aligns with a study from Saudi Arabia[16]. However, it contradicts the results of a Malaysian study, which reported no association between age and adherence levels[17]. Our observations suggest that lower adherence in the elderly population, as assessed by the ACDS, may be attributed to their tendency to forget to take medications, adjust dosages based on how they feel, and have less conviction about the necessity of following all recommended medications.

Moreover, sociodemographic factors, such as holding a vocational or university degree, were found to significantly influence medication adherence. Almarwani A’s study indicated that medication adherence could vary significantly according to the patient’s education level and could predict the degree of adherence[16]. In our study, we also found that married and non-smoker patients exhibited better adherence to their medications, although statistical significance was not achieved for these variables. This contrasts with the findings of Kosobucka *et al*, which reported that unmarried and smoking patients had better medication adherence[5].

In our study, we observed a critical influence of clinical parameters on the ACDS score. Patients without endocrine disorders such as diabetes mellitus and obesity, those with lipid disorders, those taking anti-ischemic agents, lipid-lowering agents, anti-hypertensive medications, those on 3–5 drugs, and those who underwent PCI or CABG demonstrated significantly better adherence to therapeutic recommendations.

Our study found that patients with hypertension exhibited better medication adherence than those without hypertension, although this finding did not reach statistical significance. This result contrasts with a Malaysian study that reported about three-quarters of hypertensive patients were non-adherent to their medications[18]. Additionally, a significant association was found between having a lipid disorder and medication adherence, which opposes the findings of the Malaysian study[18]. This suggests that these patients diligently follow their treatment regimens, monitor their conditions, make necessary lifestyle changes, and seek professional help when needed. Furthermore, it highlights the critical role of our physicians, who provide comprehensive education and detailed instructions on medication administration, significantly enhancing patients’ adherence to prescribed treatments. In contrast, we found a negative correlation between adherence level and the presence of endocrine disorders, including diabetes and obesity. Diabetic and obese patients were less adherent to their medications, consistent with the Malaysian study, which reported that patients with comorbidities are less likely to adhere to their medications[18].

Among medication classes, there was a positive correlation between the use of anti-ischemic, lipid-lowering, and anti-hypertensive medications and adherence levels. Patients incorporating these three classes into their regimen were more adherent than those who did not. Adherence to these medication classes plays a critical role in preventing recurrent ACS and associated complications. Two studies have reported that patients taking both lipid-lowering and anti-hypertensive medications concomitantly tend to have higher adherence levels compared to those taking only one type of medication^[19,20]^. In our study, patients taking five and less classes of drugs demonstrated greater adherence to their medications. This finding aligns with other studies^[21,22]^. Additionally, this study found that ACS patients who underwent PCI tended to have higher adherence levels than those who did not undergo PCI. This is consistent with the findings of Cao Jiaoyu[22] but contrasts with the findings of Kosobucka Agata[5] which demonstrated that patients undergoing PCI were less adherent to their treatment regimen.

Consistent with findings from other studies, forgetfulness was the most frequently reported reason for medication non-adherence among patients^[17,21]^. We believe that age-related factors contribute to this issue. Common causes such as cognitive decline, sensory impairments, and emotional factors like anxiety or depression contribute to an increased likelihood of forgetting medications. Implementing targeted interventions such as pill organizers, reminder systems, and caregiver support is essential in improving adherence among this population. Additionally, lack of follow-up (40%) was negatively correlated with adherence levels. Studies have shown that patients who have regular interactions with their healthcare providers, including frequent doctor visits, are more likely to adhere to their medication regimens[16]. Regular follow-up can improve patients’ understanding of their treatment plans, provide necessary support, and increase their motivation for medication adherence. Lack of knowledge and perceived improved health were also identified as reasons for non-adherence among our study patients, which aligns with findings from a study conducted in the United Arab Emirates[21].

## Strengths and limitations

The search for methods to assess adherence that are both effective and easy to apply remains challenging. However, this study utilized one of the most recently validated questionnaires to assess medication adherence levels among ACS patients. This is the first study in Iraq and one of the few in the Middle East to evaluate medication adherence in ACS patients.

The study does have some limitations. Firstly, patient self-reporting through questionnaires increases the risk of erroneous information and bias. Secondly, data collection was limited to a single hospital and cardiac center in Duhok, Iraqi Kurdistan, which may limit generalizability. However, these two facilities are the primary settings in Duhok that provide cardiac services to more than one and half millions of inhabitants. Lastly, the cross-sectional design of the study only allows for the measurement of variables at one point in time, and long-term follow-up was not performed. This design makes it difficult to establish causality and capture changes over time. Despite these limitations, the study’s sample size was large enough to evaluate the expected differences and associations between variables effectively.

## Conclusions and recommendations

Approximately only two-fifths of ACS patients in this study exhibited good adherence to their secondary prevention medications. Higher levels of adherence were significantly associated with patients aged 51–70, those with tertiary education, patients with dyslipidemia, those incorporating anti-ischemic, lipid-lowering, and anti-hypertensive medications into their regimen, those taking three to five classes of drugs, and those who had previously undergone PCI. The three most common reasons for medication non-adherence among study patients were forgetfulness and carelessness, lack of follow-up, and lack of knowledge.

Considering the study’s limitations, we recommend further research with direct verification of adherence levels and analysis of the potential impact of ACDS results on long-term clinical outcomes. Additionally, strategies should be developed to improve medication adherence and promote healthy behaviors simultaneously. To increase adherence among patients (ACS), the authors recommend the necessity to implement a combination of approaches. Conduct educational sessions and provide simple materials in order to explain treatment modalities and their importance in details. Regular follow-ups and mobile health solutions to provide frequent reminders and curb forgetfulness. Include family members and create patient support groups. Narrowing the number of medications, such as through the use of combination pills, is an example of reducing the complexities of polypharmacy. Identify specific challenges and address them with specific solutions including cheap medications.

## Data Availability

The datasets used in this study are fully available from the corresponding author upon request.

## Research registration unique identifying number (UIN)

Researchregistry.com. (researchregistry10756).
